# The influence of neonatal BCG vaccination on in vitro cytokine responses to *Plasmodium falciparum*

**DOI:** 10.1186/s12865-024-00611-5

**Published:** 2024-04-30

**Authors:** NL Messina, M Wang, EK Forbes, B Freyne, WP Hasang, S Germano, R Bonnici, F Summons, K Gardiner, S Donath, R Gordon, SJ Rogerson, N Curtis

**Affiliations:** 1 Murdoch Children’s Research Institute, Royal Children’s Hospital, Parkville, Australia; 2 The University of Melbourne, Parkville, Australia; 3 Tsinghua University, Beijing, China; 4 The Royal Children’s Hospital Melbourne, Parkville, Australia; 5 Department of Infectious Diseases, The Doherty Institute, The University of Melbourne, Melbourne, Australia; 6 Department of Medicine, The Doherty Institute, The University of Melbourne, Melbourne, Australia; 7 Department of Ophthalmology, Peking Union Medical College Hospital, Beijing, China; 8 Department of Paediatric Infectious Diseases, School of Medicine, Children’s Health Ireland at Crumlin, University College Dublin, Dublin, Ireland

**Keywords:** Neonate, Malaria, Cytokine, Immunity, Plasmodium falciparum, BCG, Non-specific effects

## Abstract

**Background:**

Bacillus Calmette–Guérin (BCG) vaccination has off-target protective effects against infections unrelated to tuberculosis. Among these, murine and human studies suggest that BCG vaccination may protect against malaria. We investigated whether BCG vaccination influences neonatal in vitro cytokine responses to *Plasmodium falciparum*. Blood samples were collected from 108 participants in the Melbourne Infant Study BCG for Allergy and Infection Reduction (MIS BAIR) randomised controlled trial (Clinical trials registration NCT01906853, registered July 2013), seven days after randomisation to neonatal BCG (*n* = 66) or no BCG vaccination (BCG-naïve, *n* = 42). In vitro cytokine responses were measured following stimulation with *P. falciparum-*infected erythrocytes (PfIE) or *E. coli*.

**Results:**

No difference in the measured cytokines were observed between BCG-vaccinated and BCG-naïve neonates following stimulation with PfIE or *E. coli*. However, age at which blood was sampled was independently associated with altered cytokine responses to PfIE. Being male was also independently associated with increased TNF-a responses to both PfIE and *E. coli*.

**Conclusion:**

These findings do not support a role for BCG vaccination in influencing in vitro neonatal cytokine responses to *P. falciparum*. Older neonates are more likely to develop *P. falciparum*-induced IFN-γ and IFN-γ-inducible chemokine responses implicated in early protection against malaria and malaria pathogenesis.

**Supplementary Information:**

The online version contains supplementary material available at 10.1186/s12865-024-00611-5.

## Introduction

Malaria remains one of the most important infectious diseases globally with 249 million cases and 608,000 deaths in 2022 [1]. Approximately 77% of deaths occurred in children under 5 years of age, with the greatest burden in those under 3 years old [1]. Of the five *Plasmodium* species causing malaria, *P. falciparum* is responsible for nearly all of the malaria deaths [1].

Bacillus Calmette-Guérin (BCG) is a live-attenuated vaccine that protects against tuberculosis (TB) and other mycobacterial infections [2]. BCG vaccination reduces all-cause infant mortality by approximately half in high-mortality settings [3] by decreasing deaths from infections other than TB [4]. These beneficial off-target (heterologous) effects are proposed to result from BCG-induced immunomodulation [5] including through induction of innate immune memory (trained immunity) in monocytes and natural killer (NK) cells [6].

Several studies have suggested a link between BCG vaccination and protection against malaria. A BCG-vaccination scar is associated with a significantly lower malaria-specific mortality rate (MR 0.32, 95% confidence intervals (CI) 0.13–0.76) in Guinea-Bissau [7]. Similarly, an observational study across 13 sub-Saharan African countries with 34,200 children under five years old found that BCG vaccination was associated with a lower malaria prevalence (adjusted odds ratio (aOR) 0.94, 95% CI 0.9 to 0.98) [8]. Randomised controlled trials in Guinea-Bissau have found that the protective effect of BCG vaccination at birth on neonatal mortality is seasonal and coincides with the malaria incidence pattern [9]. In BCG-vaccinated adults infected with *P. falciparum* in a human challenge model, BCG vaccination increased immune cell activation but also increased severity of malaria symptoms [10]. It has also been known since the 1970s that BCG can also protect mice in a variety of murine malaria models [11–13].

Despite the evidence for a potential role of BCG in protection against malaria in infants, little is understood about the underlying immunological mechanisms. BCG vaccination biases towards a Th1/Th17 response, and it has been proposed that this leads to greater production of pro-inflammatory cytokines during the blood-stage infection and consequent increased killing of infected red blood cells [8]. However, BCG vaccination also induces memory in innate immune cells through trained immunity which might also influence anti-pathogen responses [6, 14, 15]. To explore the potential mechanism, we investigated whether neonatal BCG vaccination influences neonatal cytokine responses to *P. falciparum* in malaria-naïve infants born in Melbourne, Australia.

## Materials and methods

### Study sites and participants

Participants were a subset of infants recruited from The Melbourne Infant Study: BCG for the prevention of allergy and infection (MIS BAIR) [16] in which Australian-born neonates were randomised to vaccination with BCG-Denmark 0.05 mL intradermally or no BCG vaccination (Clinical trials registration NCT01906853 (www.clincaltrials.gov), registered July 2013) in the first 10 days of life. The inclusion and exclusion criteria for MIS BAIR are described elsewhere [16]. MIS BAIR participants were invited to have a 7-day study home visit to provide a blood sample, which was stored as previously described [17]. Inclusion criteria for cytokine analysis were: (i) blood sample collection from May 2015 to February 2016, (ii) sufficient blood for all stimulants, or (iii) participant provided sample in previous study [17]. Exclusion criteria were suspected perinatal sepsis and receipt of blood products. As hepatitis B vaccine is routinely given at birth in Australia and may influence the off-target immune response following BCG vaccination [18], neonates who did not receive the hepatitis B vaccine were also excluded. All infants had a full-blood examination at birth and none had a white blood cell count < 2000/mm^3^.

### Culture and preparation of *Plasmodium falciparum* parasites

Frozen erythrocytes were infected with laboratory-adapted *P. falciparum* line CS2 mid-late stage trophozoites (*P. falciparum*-infected erythrocytes; PfIE) [19]. PfIE were thawed by stepwise addition of 3.5% NaCl in PBS. PfIE and uninfected erythrocytes (UE) were opsonised with 45 µg /ml rabbit anti-human IgG (MP Biomedicals) per 1.65 × 10^7^ erythrocytes.

### Whole blood stimulation assay

Whole blood samples, diluted 1:1 in RPMI 1640 with GlutaMAX™ (Gibco), were added to U-bottom wells containing 3 × 10^5^ PfIE/ml, 3 × 10^5^ UE/ml, 1 × 10^6^ colony-forming units (CFU)/ml heat-killed *E. coli* [17] or RPMI alone (unstimulated). Following 24-hour incubation (37**°**C; 5% CO_2_:air), supernatants were harvested and stored at -80**°**C until cytokine analysis.

### Cytokine quantitative analysis

Interleukin (IL)-10 and interferon gamma (IFN-γ) were quantified by ELISA (Mabtech). IL-1β, tumor necrosis factor alpha (TNF-α), IL-6, IL-8, interferon gamma induced protein 10 (IP-10), macrophage inflammatory protein 1 (MIP-1α), monocyte chemoattractant protein 1 (MCP-1), monokine induced by interferon gamma (MIG) and granulocyte-macrophage colony-stimulating factor (GM-CSF) were quantified by multiplex bead assay (Bio-Rad) using a xMAP Luminex 200 Bioanalyser. Stimulations and cytokine analyses were done blind to randomisation group.

### Statistical analysis

Statistical analysis was done using Stata v13.1 and figures created using GraphPad Prism v5.0.

Cytokine results below the lower limit of detection were assigned half the lowest detectable value. Prior to analysis, all cytokine data were log transformed. Stimulation was confirmed by comparing *E. coli* to RPMI responses, and PfIE responses to UE responses, by sign test of matched pairs. Linear regression was done to determine the effect of (i) BCG vaccination, (ii) age at blood draw (< 10 days or ≥ 10 days of age) and (iii) infant’s sex on cytokine production in response to each stimulant. The log transformed value of the unstimulated cytokine values was included as a covariate in regression analyses for UE and *E. coli* responses; and UE was included as a covariate for PfIE responses. This approach was used as per our previous studies [17, 20] to account for variability in unstimulated samples and to avoid the need for subtraction of unstimulated cytokine values. Multivariable linear regression was also done to test the models for confounding and interaction.

The study was approved by the research ethics committees of the Royal Children’s Hospital (HREC 33025) and the Mercy Hospital for Women (HREC R12/28).

## Results

### Participants

Blood samples were collected from 108 neonates at 9.4 days of age (range 4–17 days). Median age at randomisation to receive BCG (66 BCG-vaccinated) or not (42 BCG-naïve) was 1.8 (range 0–9) days of age. There were no differences in characteristics between groups (Table 1).

**Table 1:** Baseline characteristics of study participants

Characteristic	Overall n (%)	BCG-vaccinated n (%)	BCG-naïve n (%)
Overall	108 (100)	66 (61.1)	42 (38.9)
Sex			
Male	63 (58.3)	40 (60.6)	23 (54.8)
Female	45 (41.7)	26 (39.4)	19 (45.2)
Gestational age, weeks, mean ± SD	39.3 ± 1.3	39.3 ± 1.4	39.2 ± 1.2
Birth weight, grams, mean ± SD	3449 ± 502.5	3446 ± 497.9	3455 ± 515.8
Mode of delivery			
Vaginal	70 (64.8)	41 (62.1)	29 (69.1)
Caesarean	38 (35.2)	25 (37.9)	13 (30.9)
Feeding			
Breast	89 (82.4)	55 (83.3)	34 (80.9)
Formula	3 (2.8)	1 (1.5)	2 (4.8)
Combination	16 (14.8)	10 (15.2)	6 (14.3)
Maternal BCG vaccination			
Yes	25 (23.1)	15 (22.7)	10 (23.8)
No	76 (70.4)	47 (71.2)	29 (69.1)
Not reported	7 (6.5)	4 (6.1)	3 (7.1)
Age at blood collection, days			
Mean ± SD	9.4 ± 2.3	9.5 ± 2.4	9.2 ± 2.3
Median (IQR)	9 (7–11)	10 (8–11)	9 (7–11)
Age at BCG vaccination, days			
Mean ± SD	-	1.9 ± 1.6	-
Median (IQR)	-	1.2 (0.9–2.5)	-
Interval from BCG to blood collection, days, mean ± SD	-	7.5 ± 2.2	-

Data are number (%) of participants, unless otherwise stated

Abbreviations: SD, standard deviation; IQR, interquartile range

### Cytokine responses in unstimulated samples

There was a high degree of interindividual variability in cytokine responses, as seen in previous studies [17, 20, 21] (Supplementary Fig. 1). Stimulation was effective, with higher responses observed for all cytokines following *E. coli* compared to RPMI stimulation, and PfIE compared to UE or RPMI stimulation (Supplementary Fig. 1).

In samples exposed to RPMI medium only (unstimulated samples), there was no difference in cytokine or chemokine responses between BCG-vaccinated and unvaccinated neonates. However, when samples were exposed to UE, BCG-vaccinated neonates showed decreased production of the pro-inflammatory cytokines IL-6 and IL-1β (Table 2).

**Table 2:** The effect of BCG vaccination on unstimulated cytokine responses

	RPMI		Uninfected erythrocytes		
Cytokine	GMR^a^ (95% CI)	*p*-value^b^	GMR^a^ (95% CI)	*p*-value^b^	
IFN-γ	1.6 (0.7–3.3)	0.24	1.3 (0.7–2.3)	0.41	
TNF-α	1.0 (0.8–1.3)	0.96	0.94 (0.7–1.2)	0.65	
IL-6	0.7 (0.3–1.4)	0.32	0.6 (0.3-1.0)	**0.05**	
IL-1β	0.6 (0.3–1.6)	0.34	0.4 (0.2–0.9)	**0.04**	
IL-10	1.1 (0.51–2.5)	0.76	1.2 (0.6–2.5)	0.59	
MIG	0.84 (0.7–1.1)	0.13	0.8 (0.7–1.1)	0.12	
IL-8	0.8 (0.3–2.1)	0.68	0.8 (0.5–1.3)	0.34	
IP-10	1.2 (0.7-2.0)	0.57	0.7 (0.4–1.2)	0.16	
MCP-1	1.0 (0.4–2.7)	0.98	0.9 (0.5–1.8)	0.76	
MIP-1α	0.8 (0.4–1.6)	0.50	0.7 (0.4–1.1)	0.13	
GM-CSF	1.0 (0.4–2.6)	0.94	1.3 (0.5–3.7)	0.57	

^a^ Univariate analysis for the effect of BCG vaccination following stimulation with RPMI or uninfected erythrocytes. GMR > 1.0 indicates cytokine levels were higher in BCG-vaccinated neonates compared to BCG-naive neonates

^b^ p-value < 0.05 depicted in bold

Abbreviations: 95% CI, 95% confidence interval; GMR, geometric mean ratio

### Effect of BCG vaccination

**Fig. 1:**
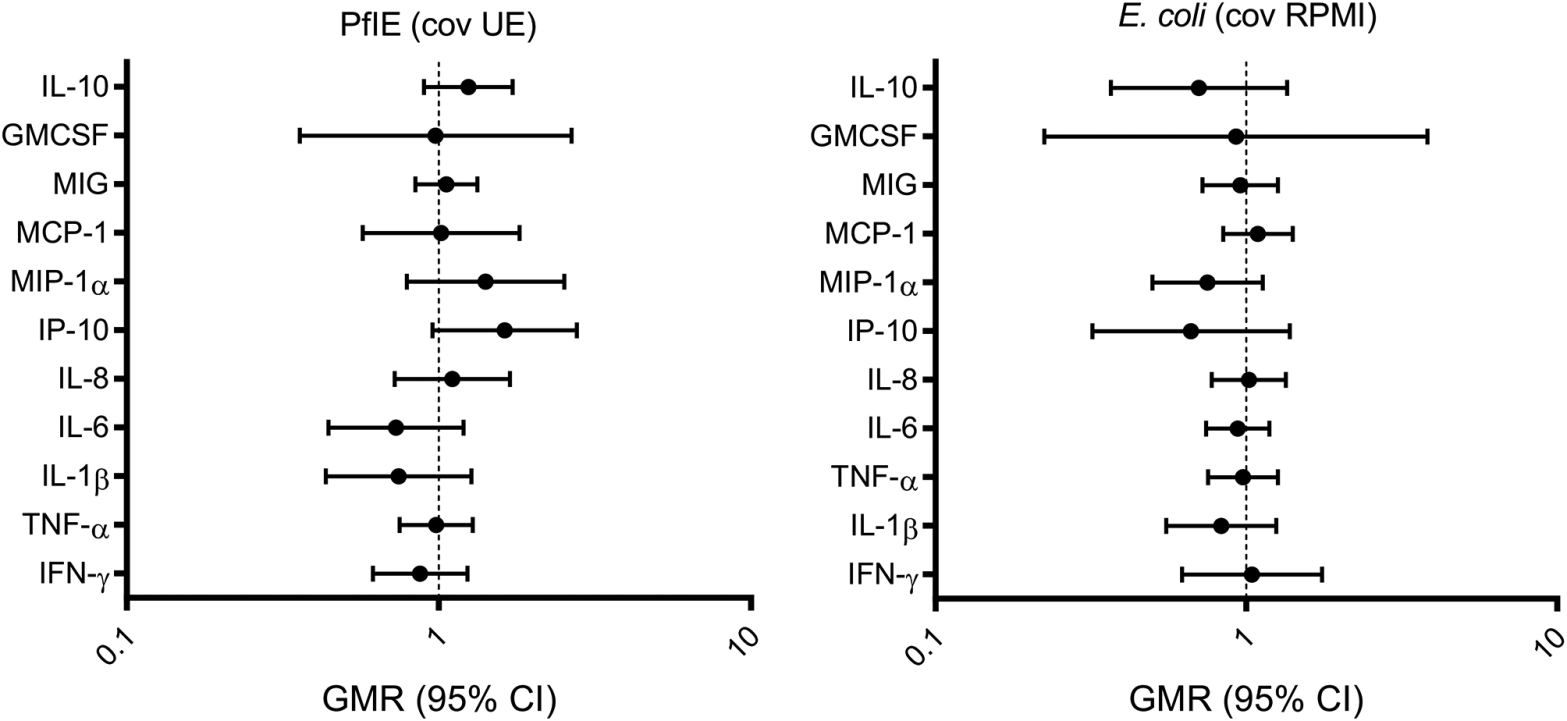
The effect of neonatal BCG vaccination on cytokine responses A geometric mean ratio (GMR) of > 1.0 indicates cytokine levels were higher in BCG-vaccinated neonates compared to BCG-naive neonates. Responses to unstimulated samples were used as a covariate (cov) in the linear regression model to avoid the need for subtraction of unstimulated cytokine values (UE for PfIE and RPMI for *E. coli*).

Following stimulation with PfIE, cytokine responses did not significantly differ between BCG-vaccinated and BCG-naïve neonates (Fig. 1, Supplementary Table 1). This remained the case after adjusting for sex and age at which blood was taken, indicating that these variables did not confound the observed responses (Supplementary Table 4).

### Effect of age

**Fig. 2:**
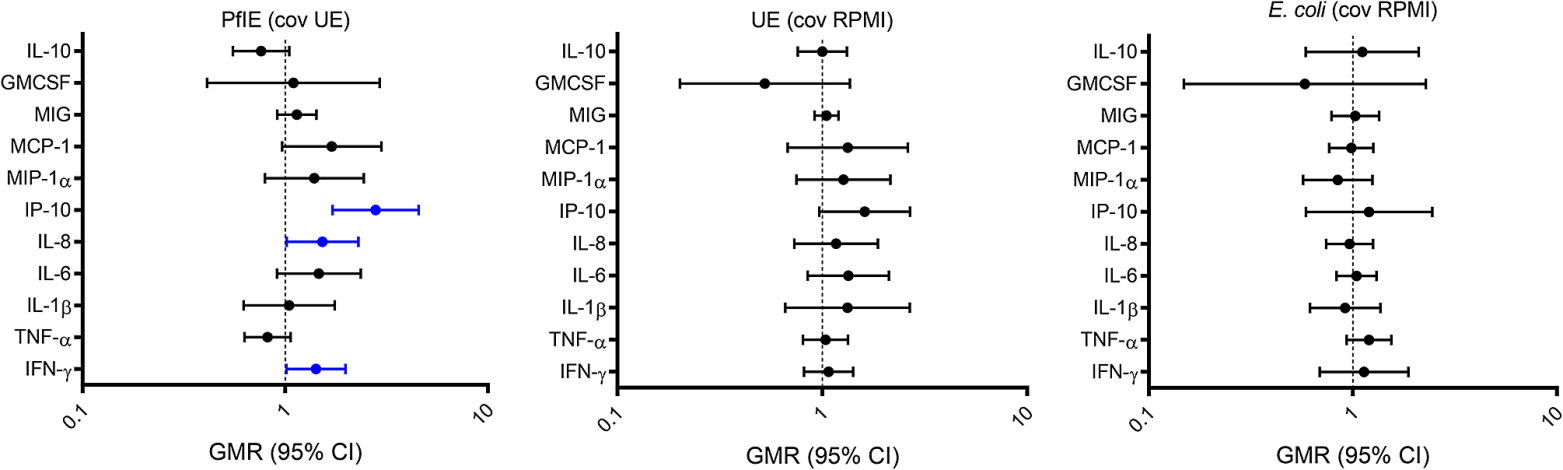
The effect of age on cytokine responses A geometric mean ratio (GMR) of > 1.0 indicates cytokine levels were higher in neonates 10 days older or over (n=53 for PfIE and UE, n=42 for *E. coli*) compared to neonates under 10 days old (n = 55 for PfIE and UE, n = 31 for E. coli). Significant results *p* < 0.05 are depicted in blue

The age of the neonate when blood was drawn independently influenced cytokine responses to *Pf*IE. Compared with neonates under 10 days old, neonates over 10 days old had increased production of IP-10 (GMR: 2.79 [95% CI 1.71–4.55], *p* < 0.01), IL-8 (GMR: 1.53 [95% CI 1.02–2.3], *p* = 0.04), and IFN-g (GMR: 1.42, [95% CI 1.0-1.99], *p* = 0.04) following stimulation with PfIE, but not *E. coli* (Fig. 2, Supplementary Table 2). Multivariable analysis to evaluate the confounding effect of neonatal BCG vaccination, sex and maternal BCG did not alter the observed effect, indicating that there was no confounding by these variables (Supplementary Table 4).

### Effect of sex

**Fig. 3:**
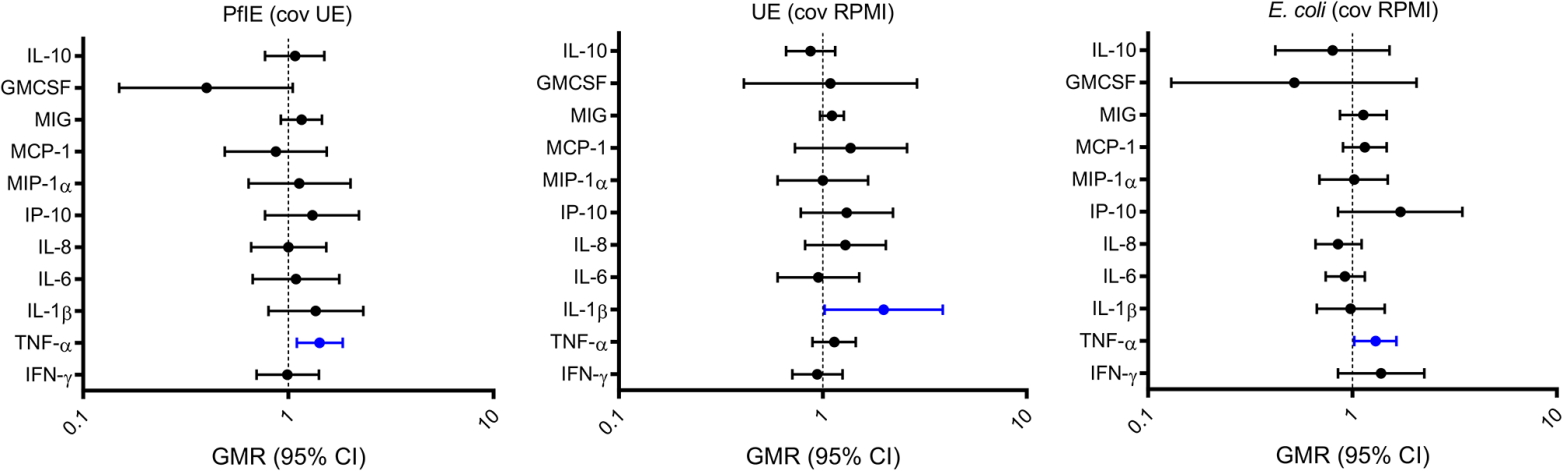
The effect of sex on cytokine responses A geometric mean ratio (GMR) of > 1.0 indicates cytokine levels were higher in males (n = 63 for PfIE and UE, n=39 for E. coli) compared to females (n =4 5 for PfIE and UE, n = 34 for E. coli). Significant results *p* < 0.05 are depicted in blue

Sex independently influenced TNF-α production following stimulation with both PfIE and *E. coli*. Compared to females, males had increased production of TNF-α in response to stimulation with PfIE (GMR: 1.42 [95% CI 1.1–1.84], *p* = 0.01) and *E. coli* (GMR: 1.3 [95% CI 1.02–1.64], *p* = 0.03) (Fig. 3, Supplementary Table 3). Multivariable analyses were done to evaluate the potential confounding effects of BCG vaccination and age, but no meaningful changes to the result were observed, ruling out confounding by these variables (Supplementary Table 4). There was no evidence of interaction between sex and neonatal BCG vaccination (data not shown).

## Discussion

### Influence of BCG vaccination

The outcome following *Plasmodium* spp. infection is a balance between cytokine-mediated pathogen eradication and damaging inflammatory immune responses [22]. During blood-stage infection, pro-inflammatory cytokines such as IFN-γ, TNF-α, IL-6 and IL-8 are crucial for controlling the growth of parasites. However, these cytokines are also associated with malaria pathology and mortality [22]. As BCG vaccination alters infant cytokine responses to other non-TB pathogens [17, 23, 24], we investigated neonatal *in-vitro* cytokine responses to *P. falciparum-*infected erythrocytes. Overall, we found that neonatal BCG vaccination did not alter early cytokine responses to *P. falciparum* infected erythrocytes in vitro. This finding is consistent with our previous study that found blood samples taken from BCG-vaccinated and BCG-naïve neonates approximately 7 days after vaccination respond differentially to only selected in vitro stimulants [17]. In that study, changes in cytokine responses were observed in BCG-vaccinated neonates following stimulation with *S. pneumoniae, E. coli, L. monocytogenes, C. albicans*, PEPG and R848, but not following stimulation with BCG, *M. tuberculosis*, group B streptococcus, *H. influenzae*, LPS or Pam3CSK4. The findings are also consistent with findings from a Danish RCT of neonatal BCG vaccination which found no effect of neonatal BCG vaccination on cytokine responses 4-days post randomisation and at 13 months of age following stimulation with a variety of stimulants [25].

These data do not rule out a role for BCG vaccination in protection from malaria. In a recent study, adults were vaccinated with BCG and underwent a controlled malaria challenge five weeks later [10]. Prior to malaria challenge there were no significant differences in in vitro NK cell or T cell responses between BCG-vaccinated and BCG-naïve individuals following stimulation with PfIE. However, post-challenge NK cells from BCG-vaccinated volunteers produced significantly more granzyme B and there were also more granzyme B-producing CD4^+^ T cells. Furthermore, following malaria challenge BCG-vaccinated individuals developed clinical symptoms at an earlier timepoint and with greater severity compared to non-BCG-vaccinated participants. Parasitaemia in BCG-vaccinated volunteers was inversely correlated with increased NK cell and monocyte activation [10]. Both the clinical and post-infection in vitro findings indicate that BCG vaccination altered the immune response to *P. falciparum* infection, despite no detectable differences in vitro prior to the malaria challenge.

BCG may protect infants from malaria through a mechanism not investigated here, such as altering the immune response to development of parasites in the liver, an effect that could not be investigated in these experiments. It has also been hypothesised that neonatal BCG vaccination leads to reduced neonatal deaths by ameliorating the detrimental immunological effects of maternal malaria infection during pregnancy, rather than by directly reducing malaria infection or malaria-associated pathology in infants [9].

### Influence of neonatal age

This study found that neonates over 10 days old had increased production of IP-10, IL-8 and IFN-γ in response to *P. falciparum* stimulation. These findings are consistent with the dynamic changes that occur during the first week of life [21]. A cohort study of 30 Gambian infants found that IP-10 and IFN-γ increase during the first seven days of life, while IL-10 and IL-6 decrease [21].

Low IFN-γ production following *in-vitro Plasmodium* spp. stimulation is associated with increased risk of parasitemia and clinical malaria [26, 27], as well as more frequent and rapid *Plasmodium* spp. re-infection in former malaria patients [28]. The chemokines IL-8, IP-10 and MIG are associated with severe and cerebral malaria purportedly by reducing the integrity of the blood-brain barrier and promoting T cell-mediated tissue damage [29, 30]. However, one murine study showing BCG-induced protection against *Plasmodium* spp. found that after infection, splenocytes from BCG-vaccinated mice had increased transcription of these chemokines compared to BCG-naïve mice [13]. Moreover, murine studies suggest that during the liver stage of infection, type I IFN-induced IP-10 and MIG promote NK and CD8^+^ T cell-mediated protection against malaria [30, 31]. Therefore, rather than being pathogenic, these chemokines might be protective by recruiting cytotoxic T lymphocytes.

Overall, our findings suggest that older neonates might develop a more robust immune response to infection with *Plasmodium* species, which may be associated with clearance of parasites, but also potentially greater pathology.

### Influence of sex

Sex is known to influence the immune response to both vaccines and infections [32, 33] and infant males have greater pro-inflammatory responses than females following stimulation with LPS or mitogens [34]. There is some evidence that sex may influence risk of malaria in infancy, with female infants having higher incidence of malaria infection during the first two years of life, following intermittent preventative treatment of malaria in pregnancy at four week intervals [35]. Here, we found that among neonates, males produced more TNF-α than females in response to both *P. falciparum* and *E. coli* stimulation. This finding is partially consistent with our previous study that found that male neonates produced more TNF-α and IFN-γ than female neonates in response to heterologous stimulants seven days post-randomisation, regardless of vaccination group [18]. It is possible that greater production of TNF-α in male infants may play a role in control of parasitaemia in male neonates relative to female neonates, and this requires further investigation.

In contrast to other studies, our study did not find a differential effect of BCG vaccination between male and female neonates. A study in Guinea-Bissau found that among low-birth weight babies, boys had a higher mortality rate than girls during the first week and that BCG had a significant beneficial effect in boys during the first week, reducing the mortality 3-fold. In girls, there was limited benefit of BCG during the first week, but BCG had a significantly beneficial effect on mortality during weeks 2–4 [36]. That study did not investigate the underlying immune responses responsible for the differential response to BCG between males and female neonates, but the findings suggest a differential response.

### Limitations

A limitation of this study was the substantial variation between participants in the cytokine responses observed. This is consistent with previous studies [17] and is expected in neonates [21]. The use of whole blood, which contains a variety of cellular components as well as antibodies [37], may have contributed to the observed variability. To account for this, we included the unstimulated value for each cytokine as a covariate in the linear regression analyses, including using UE as a covariate in analyses of responses to infected erythrocytes.

The study is also limited by the relatively small sample size and the use of multiple statistical tests which could result in both type 1 and type 11 error. To aid interpretation all results are presented with confidence intervals and emphasis has not been place on p-values [38].

Further limitations are that immune responses were investigated in neonates whose immune systems are immature and rapidly developing [21], and that immune responses were investigated only seven days post vaccination. While an effect of BCG on cytokine responses has been observed seven days post vaccination previously [17], the early time-point could have contributed to the lack of an observed effect of BCG. Furthermore, given the liver-stage of the *P. falciparum* life cycle lasts about seven days following the bite of an infected mosquito, neonates will only be exposed to blood-stage malaria through congenital infection, when their immune response would also be influenced by *in utero* exposure to malaria antigens and maternal antibodies. This limits the real-world interpretation of the findings.

Due to the limited blood volume available from neonates, only one parasite line could be tested in this study. Further studies are needed to determine whether similar responses are observed using other parasite lines. Moreover, future multi-omics studies mapping the immune landscape will be valuable in identifying the molecular pathways that underpin observed cytokine responses [39].

This study was done in Australia in neonates who were therefore not exposed to malaria. This had the advantage that there was no prior exposure to malaria during pregnancy that might influence the response between individuals. However, further studies to elucidate the cytokine response and effect of BCG in protection against malaria in malaria-endemic countries would be worthwhile.

## Conclusions

In conclusion, this study in neonates in Australia did not find an effect of BCG on in vitro immune responses to *P. falciparum*. The study does provide evidence that older neonates and boys develop a greater pro-inflammatory response to *P. falciparum* than younger neonates or girls.

### Electronic supplementary material

Below is the link to the electronic supplementary material.

Supplementary Material 1

## Data Availability

The datasets generated during and analyzed during the current study are available from the corresponding author on reasonable request.
