# Supplementary material for: The influence of neonatal BCG vaccination on in vitro cytokine responses to Plasmodium falciparum
Source: BMC Immunol. 2024 Apr 30;25:24. doi: 10.1186/s12865-024-00611-5 (PMC11059926; doi:10.1186/s12865-024-00611-5)
Supplement: Supplementary file 1 — Supplementary Material 1 [file 12865_2024_611_MOESM1_ESM.pdf]

# Supplementary Information

## Supplementary Figure 1. Immune response profiles (pg/mL) and stimulation index

Cytokine levels in all participant samples after stimulation with RPMI (Nil), E. Coli, uninfected erythrocytes or Plasmodium falciparum-infected erythrocytes. Black line represents median values and error bars represent interquartile range. ** p<0.01, *** p<0.001 by sign test of matched pairs.

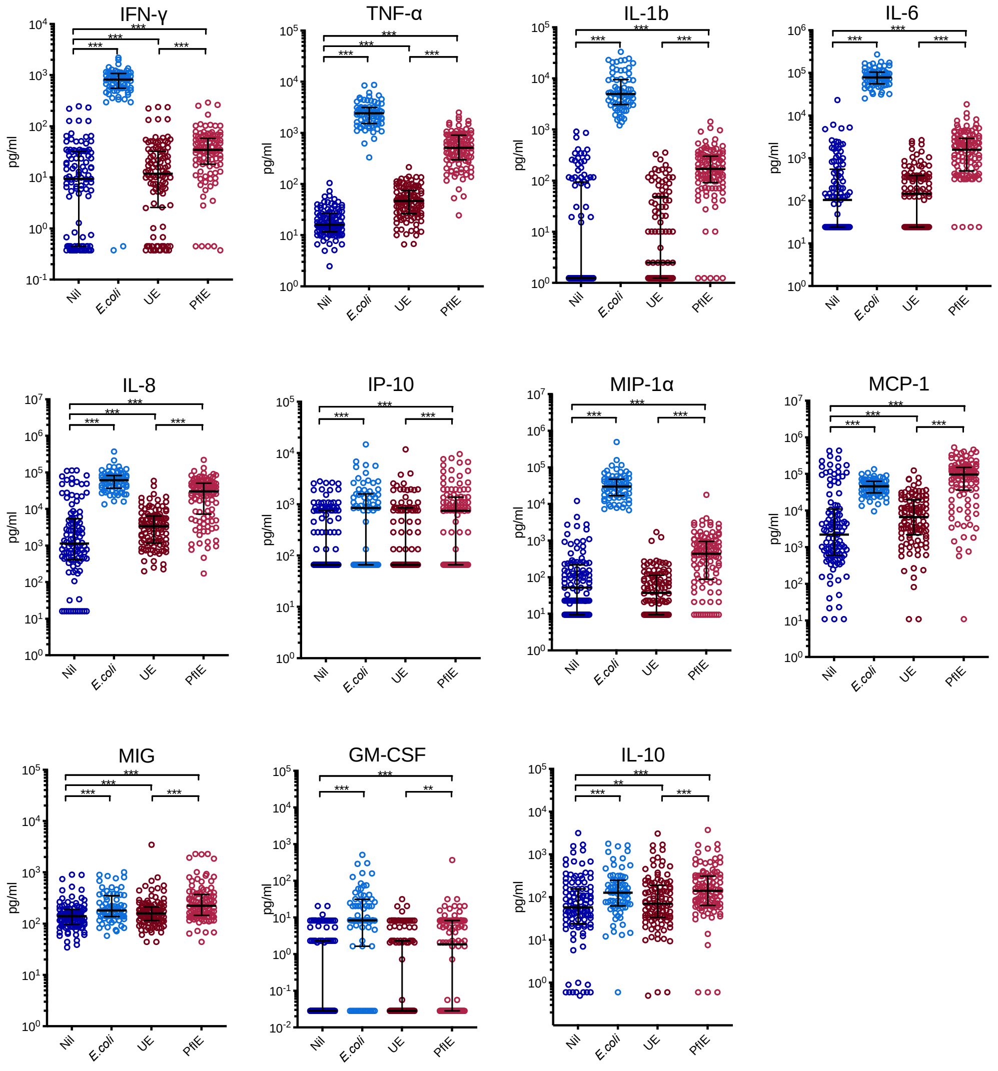

**Supplementary Table 1. The effect of BCG vaccination on cytokine responses**

| | PfIE (cov UE) | | *E. coli* (cov RPMI) | |
|---|---|---|---|---|
| **Cytokine** | **GMR[a] (95% CI)** | **p-value[b]** | **GMR[a] (95% CI)** | **p-value[b]** |
| IL-10 | 1.24 (0.9-1.7) | 0.19 | 0.70 (0.4-1.4) | 0.29 |
| GM-CSF | 0.98 (0.4-2.7) | 0.96 | 0.93 (0.2-3.8) | 0.92 |
| MIG | 1.06 (0.8-1.3) | 0.62 | 0.96 (0.7-1.3) | 0.75 |
| MCP-1 | 1.02 (0.6-1.8) | 0.95 | 1.09 (0.8-1.4) | 0.5 |
| MIP-1α | 1.41 (0.8-2.5) | 0.24 | 0.75 (0.5-1.1) | 0.17 |
| IP-10 | 1.63 (0.96-2.8) | 0.07 | 0.66 (0.3-1.4) | 0.27 |
| IL-8 | 1.10 (0.7-1.7) | 0.64 | 1.02 (0.8-1.3) | 0.89 |
| IL-6 | 0.73 (0.4-1.2) | 0.21 | 0.94 (0.7-1.2) | 0.60 |
| IL-1β | 0.74 (0.4-1.3) | 0.28 | 0.83 (0.6-1.2) | 0.37 |
| TNF-α | 0.98 (0.7-1.3) | 0.89 | 0.98 (0.8-1.3) | 0.85 |
| IFN-γ | 0.87 (0.6-1.2) | 0.43 | 1.04 (0.6-1.8) | 0.87 |

[a] Multivariate analysis for the effect of BCG vaccination following stimulation with PfIE (covariate UE) or E. coli (covariate RPMI). GMR >1.0 indicates cytokine levels were higher in BCG-vaccinated neonates compared to BCG-naive neonates.

[b] p-value <0.05 depicted in bold

95% CI, 95% Confidence interval; GMR, Geometric mean ratio

**Supplementary Table 2. The effect of age on cytokine responses.**

| | PfIE (cov UE) | | *E. coli* (cov RPMI) | | UE (cov RPMI) | |
|---|---|---|---|---|---|---|
| **Cytokine** | **GMR[a] (95% CI)** | **p-value[b]** | **GMR[a] (95% CI)** | **p-value[b]** | **GMR[a] (95% CI)** | **p-value[b]** |
| IL-10 | 0.76 (0.6-1.05) | 0.09 | 1.11 (0.6-2.1) | 0.74 | 1.00 (0.8-1.3) | 1.00 |
| GM-CSF | 1.10 (0.4-2.9) | 0.85 | 0.58 (0.1-2.3) | 0.43 | 0.52 (0.2-1.4) | 0.18 |
| MIG | 1.14 (0.9-1.4) | 0.25 | 1.03 (0.8-1.3) | 0.84 | 1.05 (0.9-1.2) | 0.48 |
| MCP-1 | 1.70 (0.97-2.98) | 0.07 | 0.98 (0.8-1.3) | 0.89 | 1.33 (0.7-2.6) | 0.40 |
| MIP-1α | 1.39 (0.8-2.4) | 0.25 | 0.84 (0.6-1.2) | 0.39 | 1.27 (0.7-2.1) | 0.37 |
| IP-10 | 2.79 (1.7-4.6) | **0.00** | 1.20 (0.6-2.5) | 0.61 | 1.61 (1.0-2.7) | 0.07 |
| IL-8 | 1.53 (1.02-2.3) | **0.04** | 0.96 (0.7-1.3) | 0.78 | 1.17 (0.7-1.9) | 0.51 |
| IL-6 | 1.47 (0.9-2.4) | 0.11 | 1.04 (0.8-1.3) | 0.71 | 1.34 (0.8-2.1) | 0.21 |
| IL-1β | 1.05 (0.6-1.7) | 0.86 | 0.92 (0.6-1.4) | 0.67 | 1.33 (0.7-2.7) | 0.42 |
| TNF-α | 0.82 (0.6-1.1) | 0.13 | 1.20 (0.9-1.5) | 0.16 | 1.04 (0.8-1.3) | 0.78 |
| IFN-γ | 1.42 (1.01-1.99) | **0.04** | 1.14 (0.7-1.9) | 0.61 | 1.07 (0.8-1.4) | 0.61 |

[a] Multivariate analysis for the effect of age following stimulation with PfIE (covariate UE) or E. coli (covariate RPMI). GMR >1.0 indicates cytokine levels were higher in neonates over 10 days old as compared to neonates under ten days old.

[b] p-value <0.05 depicted in bold

95% CI, 95% Confidence interval; GMR, Geometric mean ratio

**Supplementary Table 3. The effect of sex on cytokine responses.**

| | **PfIE (cov UE)** | | ***E. coli* (cov RPMI)** | | **UE (cov RPMI)** | |
|---|---|---|---|---|---|---|
| **Cytokine** | **GMR[a] (95% CI)** | **p-value[b]** | **GMR[a] (95% CI)** | **p-value[b]** | **GMR[a] (95% CI)** | **p-value[b]** |
| IL-10 | 1.08 (0.8-1.5) | 0.66 | 0.80 (0.4-1.5) | 0.48 | 0.87 (0.7-1.2) | 0.33 |
| GM-CSF | 0.40 (0.2-1.1) | 0.06 | 0.52 (0.1-2.1) | 0.35 | 1.09 (0.4-2.9) | 0.87 |
| MIG | 1.16 (0.9-1.5) | 0.20 | 1.13 (0.9-1.5) | 0.35 | 1.11 (1.0-1.3) | 0.12 |
| MCP-1 | 0.87 (0.5-1.5) | 0.63 | 1.15 (0.9-1.5) | 0.27 | 1.37 (0.7-2.6) | 0.32 |
| MIP-1α | 1.13 (0.6-2.0) | 0.67 | 1.02 (0.7-1.5) | 0.93 | 1.00 (0.6-1.7) | 0.99 |
| IP-10 | 1.31 (0.8-2.2) | 0.32 | 1.72 (0.9-3.5) | 0.13 | 1.31 (0.8-2.2) | 0.30 |
| IL-8 | 1.00 (0.7-1.5) | 0.99 | 0.85 (0.7-1.1) | 0.23 | 1.29 (0.8-2.0) | 0.27 |
| IL-6 | 1.09 (0.7-1.8) | 0.73 | 0.92 (0.7-1.2) | 0.47 | 0.95 (0.6-1.5) | 0.84 |
| IL-1β | 1.36 (0.8-2.3) | 0.26 | 0.98 (0.7-1.4) | 0.92 | 1.99 (1.0-3.9) | **0.04** |
| TNF-α | 1.42 (1.1-1.8) | **0.01** | 1.30 (1.0-1.6) | **0.03** | 1.14 (0.9-1.5) | 0.31 |
| IFN-γ | 0.99 (0.7-1.41) | 0.95 | 1.38 (0.9-2.3) | 0.19 | 0.94 (0.7-1.3) | 0.65 |

[a] Multivariate analysis for the effect of sex following stimulation with PfIE (covariate UE) or E. coli (covariate RPMI). GMR >1.0 indicates cytokine levels were higher in males as compared to females.

[b] p-value <0.05 depicted in bold

95% CI, 95% Confidence interval; GMR, Geometric mean ratio

**Supplementary Table 4. Multivariable linear regression of the effect of neonatal BCG vaccination, sex and age on cytokine responses following stimulation with PfIE**

| | BCG (vaccinated vs unvaccinated) | | Age (10 days old vs under 10 days old) | | Sex (males vs females) | |
|---|---|---|---|---|---|---|
| **Cytokine** | **GMR[a] (95% CI)** | **p-value[b]** | **GMR[a] (95% CI)** | **p-value[b]** | **GMR[a] (95% CI)** | **p-value[b]** |
| IL-10 | 1.26 (0.91-1.74) | 0.17 | 0.75 (0.55-1.04) | 0.09 | 1.03 (0.74-1.44) | 0.84 |
| GM-CSF | 1.03 (0.38-2.81) | 0.95 | 0.99 (0.37-2.64) | 0.98 | 0.39 (0.15-1.06) | 0.06 |
| MIG | 1.04 (0.82-1.3) | 0.75 | 1.15 (0.92-1.44) | 0.22 | 1.17 (0.93-1.47) | 0.19 |
| MCP-1 | 0.99 (0.55-1.76) | 0.96 | 1.68 (0.95-2.99) | 0.08 | 0.93 (0.52-1.65) | 0.80 |
| MIP-1α | 1.37 (0.76-2.46) | 0.29 | 1.38 (0.78-2.44) | 0.26 | 1.15 (0.65-2.05) | 0.62 |
| IP-10 | 1.45 (0.88-2.39) | 0.14 | 2.84 (1.74-4.62) | **0.00** | 1.45 (0.89-2.36) | 0.14 |
| IL-8 | 1.07 (0.7-1.63) | 0.76 | 1.53 (1.01-2.32) | **0.05** | 1.05 (0.69-1.60) | 0.82 |
| IL-6 | 0.71 (0.43-1.16) | 0.17 | 1.52 (0.94-2.45) | 0.09 | 1.17 (0.72-1.90) | 0.53 |
| IL-1β | 0.71 (0.41-1.22) | 0.22 | 1.10 (0.65-1.84) | 0.72 | 1.42 (0.83-2.44) | 0.20 |
| TNF-α | 0.97 (0.75-1.26) | 0.82 | 0.85 (0.65-1.10) | 0.20 | 1.40 (1.08-1.82) | **0.01** |
| IFN-γ | 0.85 (0.6-1.21) | 0.37 | 1.43 (1.02-2.02) | **0.04** | 1.04 (0.73-1.47) | 0.83 |

[a] Multivariate analysis for the effect of BCG vaccination, age and sex following stimulation with P. falciparum infected erythrocytes (PfIE) using the model $Y = \beta_0 + \beta_1 BCG + \beta_2 Age + \beta_3 Sex + \beta_4 UE$.

[b] p-value <0.05 depicted in bold

95% CI, 95% Confidence interval; GMR, Geometric mean ratio
